# Canvas of Strength: A Mixed-Methods Pilot Study of a Community-Based Arts Intervention for Women Survivors of Domestic Violence in China

**DOI:** 10.3390/bs16091563

**Published:** 2026-09-03

**Authors:** Xingcen Liu, Kamal Sabran

**Affiliations:** School of the Arts, Universiti Sains Malaysia, Gelugor 11800, Penang, Malaysia; liuxingcen@student.usm.my

**Keywords:** domestic violence, pilot study, arts-based intervention, mixed methods, depression, anxiety, self-esteem, China

## Abstract

Women survivors of domestic violence often experience persistent psychological distress, yet evidence supporting community-based arts interventions remains limited, particularly in mainland China. This mixed-methods pilot study evaluated the preliminary effectiveness of Canvas of Strength, a community-based arts intervention for women survivors of domestic violence in Liaoning Province, China. Sixteen participants were recruited from a community service center and allocated to intervention (n = 8) and control (n = 8) groups. Depression, anxiety, and self-esteem were assessed at baseline, post-intervention, and six-month follow-up using standardized measures, while semi-structured interviews explored participants’ experiences. The intervention group demonstrated significant improvements across all three psychological outcomes following the intervention, with these gains largely maintained at six-month follow-up, whereas no significant changes were observed in the control group. Qualitative findings revealed enhanced emotional expression, positive cognitive reframing, improved self-perception, and strengthened interpersonal and social engagement, providing contextual support for the quantitative findings. Together, these findings suggest that Canvas of Strength shows promise as a community-based arts intervention for improving the psychological well-being of women survivors of domestic violence. Given the pilot nature and small sample size of this study, further evaluation in adequately powered trials is warranted.

## 1. Introduction

Intimate partner violence (IPV) affects approximately one in three women globally and is consistently associated with substantial psychological consequences, including depression, anxiety, post-traumatic stress disorder, and chronically impaired self-esteem (World Health Organization [WHO], 2025; Lagdon et al., 2014; White et al., 2024). Of these sequelae, diminished self-esteem warrants particular attention, as IPV exposure can adversely affect self-concept, self-respect, self-worth, and self-confidence among women experiencing IPV (Walker, 2009; Güler et al., 2022). Recovery is further complicated by trauma-associated shame, social withdrawal, and the fragmentation of self-concept that chronic victimization engenders (Herman, 1992). These consequences converge to create substantial barriers to help-seeking, as many survivors struggle to engage with conventional mental health services that depend on verbal disclosure of traumatic experience in structured clinical settings (B. Liang et al., 2005), a modality that may not adequately accommodate survivors whose experiences of trauma are difficult to verbalize (Malchiodi, 2011). Arts-based approaches may provide alternative forms of expression through which survivors can represent and explore experiences of abuse using visual imagery alongside spoken narratives (Bird, 2018). The implication is that effective support for domestic violence (DV) survivors may require modalities that do not place verbal disclosure at the center of therapeutic engagement.

In mainland China, this challenge is compounded by a research and resource landscape that has not kept pace with the documented burden of IPV. Yuan and Hesketh (2021), in a cross-sectional study of nearly 3000 women across six Chinese provinces, reported depression prevalence rates of 65.8%, 69.5%, and 75.8% among women experiencing psychological, physical, and sexual IPV, respectively, with all forms of violence associated with a more than twofold increase in the odds of depression. Yet systematic and scoping reviews confirm that Chinese IPV research has been concentrated almost exclusively on epidemiology and risk factor identification, with structured psychosocial intervention studies remaining rare (Zhao et al., 2022; Li et al., 2020). More broadly, recent evidence mapping of arts-based interventions for women survivors of DV indicates that these interventions often target individual, interpersonal, and community dimensions, whereas formal outcome evaluations remain disproportionately focused on individual psychological measures (Liu & Sabran, 2026). This gap is compounded by cultural contexts where DV carries significant stigma and community-level mental health infrastructure is limited. As a result, many survivors do not access available services even where they exist (Li et al., 2020; Overstreet & Quinn, 2013). This reinforces the case for approaches capable of reaching this population through non-clinical, community-embedded channels. Arts-based interventions have attracted increasing attention as a modality for addressing the psychological sequelae of IPV, and the theoretical rationale is well established. Creative practice provides a non-verbal channel through which traumatic experience can be externalized and processed without the immediate verbal disclosure that talking-based therapies require, a feature of particular relevance for survivors for whom language may be an insufficient or emotionally unsafe vehicle for trauma expression (Malchiodi, 2011; van der Kolk, 2014). Group delivery adds a further dimension by fostering peer solidarity and counteracting the social isolation that DV survivors commonly experience (Yalom & Leszcz, 2020). Collectively, existing empirical studies are encouraging, if methodologically heterogeneous: Aktaş Özkafacı and Eren (2020) reported significant reductions in depression, anxiety, and hopelessness following structured art psychotherapy; Sabina et al. (2023) documented improvements in self-esteem and general health in a controlled study; and qualitative work by Bird (2018) and Skop et al. (2022) has identified agency, identity reconstruction, and creative empowerment as central themes in DV survivors’ accounts of arts-based group participation. A recent scoping review of art therapy with women survivors of IPV identified rebuilding the self, symbolic storytelling, interpersonal and cultural reconnection, safe spaces, and hope as recurring therapeutic functions across the literature (Birger Sagiv & Goldner, 2025). In Chinese clinical populations more broadly, Xu et al. (2025) confirmed in a systematic review of 34 studies that arts-based approaches demonstrate effectiveness for depression and psychological distress, yet none of the included studies targeted survivors of DV. Although Canvas of Strength was designed as a community-based arts intervention rather than formal art therapy, its design is informed by, and situated in dialogue with, the broader art therapy literature on IPV recovery, particularly regarding the creation of safe relational spaces and symbolic modes of expression (Skop et al., 2022; Birger Sagiv & Goldner, 2025), as well as processes of identity reconstruction (Bird, 2018; Skop et al., 2022), while the program retained a non-clinical workshop format consistent with the cultural de-medicalization principle underlying its design.

Notwithstanding this emerging evidence base, several critical gaps remain. Most existing studies have lacked concurrent control groups, making it difficult to distinguish intervention-specific effects from spontaneous recovery or concurrent influences (Aktaş Özkafacı & Eren, 2020; Teague et al., 2006). More consequentially, follow-up data are largely absent from the literature: Vela et al. (2016) and Ikonomopoulos et al. (2017) both explicitly identified this as a core limitation of their work, and this gap extends across the field more broadly. Without follow-up evidence, it remains unclear whether arts-based interventions facilitate sustained psychological recovery or merely transient post-intervention improvements, a distinction with fundamental implications for how the value of such programs should be understood. A geographically specific gap further compounds these limitations: to our knowledge, no published study has evaluated a structured arts-based psychological intervention for women survivors of DV in mainland China and reported follow-up outcome data.

The present study was designed to address these gaps through a two-phase mixed-methods pilot study conducted at a community service center in Haicheng, Liaoning Province, China. The Canvas of Strength intervention was adapted from the evidence-based See the Triumph model (Murray et al., 2017) and delivered across eight group sessions organized into four sequential modules. It targeted depression, anxiety, and self-esteem as primary outcomes. These outcomes were assessed using validated Chinese versions of the Self-Rating Depression Scale (SDS), Self-Rating Anxiety Scale (SAS), and Rosenberg Self-Esteem Scale (RSES) at baseline, post-intervention, and six-month follow-up in a sample of 16 women (experimental group n = 8; control group n = 8). Structured behavioral observation and semi-structured follow-up interviews provided complementary process and qualitative data. Accordingly, this pilot study contributes to the existing literature in three specific respects: it represents, to our knowledge, the first evaluation of a structured arts-based group intervention for DV survivors in mainland China; it incorporates a concurrent control group absent from most comparable work; and it extends assessment to a six-month follow-up, addressing the most consequential methodological gap in the field to date.

## 2. Materials and Methods

### 2.1. Study Design

This study adopted a mixed-methods pilot design comprising two sequential phases (Creswell & Plano Clark, 2017). The first phase employed a randomized pre-test–post-test control group pilot design to examine the short-term effects of the intervention; the second combined a six-month quantitative follow-up with qualitative interviews to evaluate outcome sustainability. Sample size was not determined by formal power calculations; this study was designed to generate preliminary effect size estimates and assess intervention feasibility to inform a future confirmatory trial (Thabane et al., 2010).

### 2.2. Participants

#### 2.2.1. Setting and Recruitment

This study was conducted at the Sanyi Community Service Centre in Haicheng, Anshan, Liaoning Province, China. The community has an established administrative mediation system but lacks dedicated mental health services for DV survivors. A resource-constrained, non-clinical community setting was deliberately selected to enhance accessibility for vulnerable survivors, reduce help-seeking stigma (Malchiodi, 2011), and evaluate real-world intervention feasibility (Thabane et al., 2010). Recruitment, screening, and intervention delivery were supported by a multidisciplinary team comprising a psychiatrist (E1), a mental health counsellor (E2), a social worker (E3), and a certified expressive arts therapist (E4).

**Figure 1:**
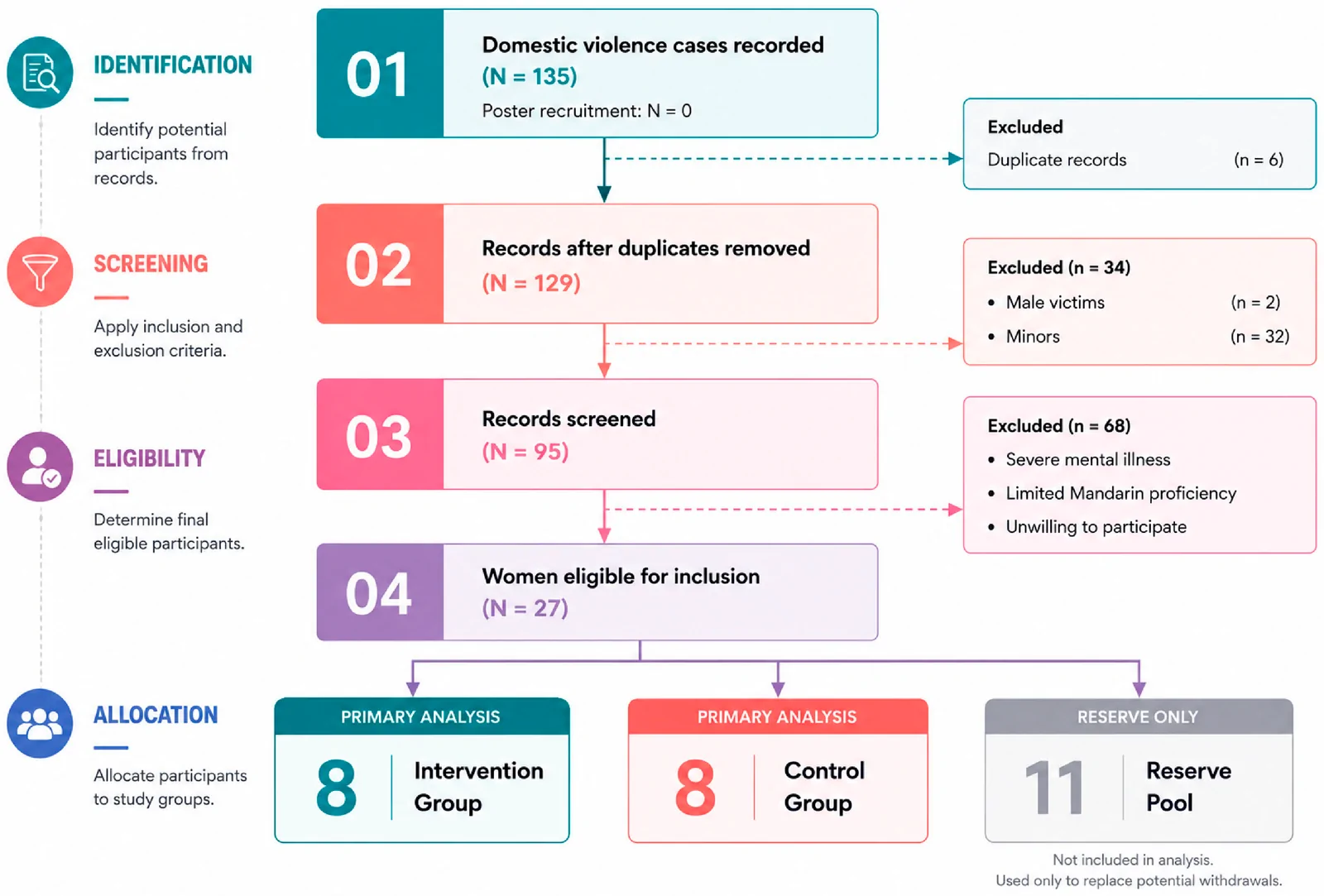
Flow diagram of participant identification, screening, and allocation.

Participants were identified through a pre-existing family dispute mediation registry maintained by the community service center as part of routine administrative practice, covering records from 1 January to 20 November 2024 (N = 135). Access to this registry, and all subsequent participant contact and screening, occurred only after ethics approval was obtained on 15 November 2024. As a supplementary recruitment strategy, posters were displayed at the local Cultural Arts Centre in Sanyi Community from 18 November to 15 December 2024. No participants were recruited through this poster-based channel. After removing duplicate records (n = 6) and excluding male victims (n = 2, excluded to maintain intervention group homogeneity and psychological safety) and minors (n = 32), 95 candidates underwent telephone screening led by the social worker (E3) with clinical risk assessment support from the study psychiatrist (E1). Through this criterion-based purposive screening process (Patton, 2015), a total of 27 women were confirmed eligible (Figure 1). Each eligible candidate was assigned a numeric identifier (1–27), and group allocation was determined through random selection from this numbered pool: 8 candidates were randomly drawn to form the experimental group (P1–P8), a further 8 were randomly drawn to form the control group (P9–P16), and the remaining 11 candidates constituted a reserve pool as an attrition buffer. A group size of eight was consistent with recommendations for therapeutic group interventions (Yalom & Leszcz, 2020) and appropriate for pilot feasibility assessment (Thabane et al., 2010).

#### 2.2.2. Inclusion and Exclusion Criteria

Inclusion criteria were: (1) adult females aged 18 years or older; (2) self-reported DV history (physical, emotional, or sexual); (3) no concurrent psychological or psychiatric treatment; and (4) sufficient Mandarin proficiency and willingness to attend all eight sessions.

Exclusion criteria were: (1) severe mental illness (schizophrenia, bipolar disorder, or severe psychotic depression); (2) severe substance dependency; (3) insufficient Mandarin proficiency; and (4) ongoing crisis requiring emergency shelter or legal protection. Psychiatric screening was conducted by the study psychiatrist (E1) using the Diagnostic and Statistical Manual of Mental Disorders, 5th edition (DSM-5) framework (American Psychiatric Association, 2013) as a safety assessment rather than a formal diagnostic procedure.

### 2.3. Intervention: The Canvas of Strength Protocol

**Figure 2:**
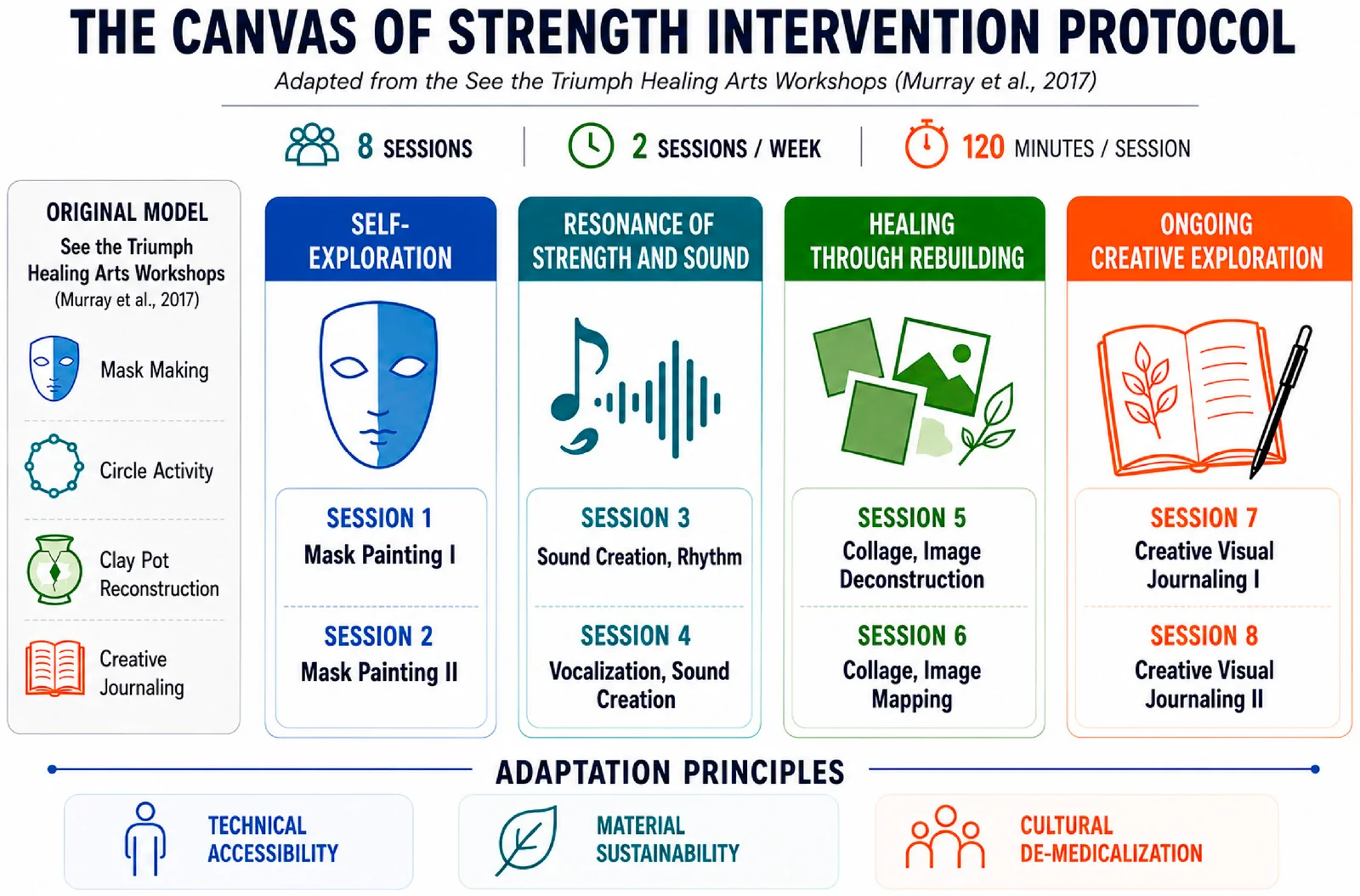
The Canvas of Strength intervention protocol, adapted from Murray et al. (2017). Eight sessions were delivered over four consecutive weeks (28 December 2024–19 January 2025), twice weekly, each lasting approximately 120 min (15 min review; 85 min art activity; 20 min reflection and sharing). The four modules were sequenced according to the progressive logic of trauma recovery—from safety establishment and self-exploration, through emotional regulation and cognitive reconstruction, to daily integration—reflecting the staged process of trauma-informed care (Herman, 1992; van der Kolk, 2014). Full session content and objectives are presented in Table 1.

The Canvas of Strength protocol was adapted from the evidence-based ‘See the Triumph’ model (Murray et al., 2017) under review by the expressive arts therapist (E4). The adaptation was guided by three principles: technical accessibility (skill-neutral activities to lower participation barriers), material sustainability (low-cost, readily available materials to support continued self-care), and cultural de-medicalization (framing the intervention as a creative workshop rather than clinical psychotherapy to reduce stigma; Malchiodi, 2011). The original model’s four core activities—mask, circle, reconstruction, and journal—each informed a corresponding adapted module, as illustrated in Figure 2.

**Table 1:** Overview of the Canvas of Strength intervention sessions and themes.

Theme	Session	Activity Title	Main Content	Activity Objective
Self-Exploration	1	Mask Painting I	Participants create personal masks using colors and patterns to express inner and outer emotions, exploring self-identity and roles.	Facilitate self-awareness and emotional externalization; support internal reflection.
	2	Mask Painting II	Additional symbolic elements are added to the masks; participants share personal stories, fostering mutual understanding and trust.	Promote self-acceptance and group trust; enhance a sense of safety and connection.
Resonance of Strength and Sound	3	Sound Creation, Rhythm	Guided by a basic rhythm, participants use simple objects to create sounds, experiencing engagement and cooperation through rhythm.	Stimulate sensory connection through sound and body; experience group support and inner strength.
	4	Vocalization, Sound Creation	Building on object-based sounds, participants are encouraged to vocalize (e.g., humming, chanting), naturally merging into group rhythm.	Encourage free vocal expression; strengthen emotional resonance and sense of belonging within the group.
Healing Through Rebuilding	5	Collage, Image Deconstruction	Using images, colors, and words, participants deconstruct and reassemble fragments of memory into a “new landscape,” engaging in a process of rebuilding with choice and agency.	Express inner emotions through visual and tactile means; experience autonomy and strength in reconstruction.
	6	Collage, Image Mapping	Continuing from the previous session, participants create a personal “map of strength” by collaging elements that evoke support, safety, or energy.	Identify and represent inner resources; enhance confidence and sense of security.
Ongoing Creative Exploration	7	Creative Visual Journaling I	Through drawing, symbols, or writing, participants document their experiences, creating a “Creative Visual Journal” to extend artistic engagement beyond the sessions.	Encourage sustained self-expression and emotional integration; provide support for ongoing self-care.
	8	Creative Visual Journaling II	Participants depict personal goals or future aspirations, expressing hope and motivation for change and growth.	Foster a positive outlook toward the future; inspire confidence and inner drive for personal development.

Note. Each of the four modules spanned two consecutive sessions. The intervention was delivered twice weekly over four weeks (eight sessions total).

The intervention was delivered by the social worker (E3), who led Modules 1, 3, and 4, and the expressive arts therapist (E4), who led Module 2. The mental health counsellor (E2) facilitated post-session emotional integration, and the study psychiatrist (E1) monitored psychological safety throughout. A Stop Rule was operative across all sessions: upon any strong adverse emotional reaction, the activity was immediately paused and a protocol activated comprising temporary withdrawal, grounding techniques, and immediate psychiatric assessment, with referral initiated if required. The Stop Rule was not activated during any of the eight sessions. The researcher performed observation and logistical roles only and did not participate in intervention delivery.

### 2.4. Measures

Depression, anxiety, and self-esteem were assessed using the Zung Self-Rating Depression Scale (SDS; Zung, 1965; Chinese norms: Wang & Wang, 1999; clinical cut-off score ≥ 53), the Zung Self-Rating Anxiety Scale (SAS; Zung, 1971; Chinese norms: Y. Liang et al., 2020; cut-off ≥ 50), and the Rosenberg Self-Esteem Scale (RSES; Rosenberg, 2015; Chinese validation: Jiang et al., 2023; higher scores indicate greater self-esteem). The RSES was selected because self-esteem impairment is a central and enduring consequence of intimate partner violence (Walker, 2009). All instruments were administered in paper-based format under the supervision of the study psychiatrist (E1) at three time points: baseline (T0: 28 December 2024), post-intervention (T1: 19 January 2025), and six-month follow-up (T2: 19 July 2025). Internal consistency reliability (Cronbach’s α) for each instrument at each time point is reported in Table 2.

### 2.5. Qualitative Data Collection

#### 2.5.1. Structured Observation Records

Behavioral changes across all eight sessions were documented using Structured Observation Records (Angrosino, 2007). The researcher maintained contemporaneous field notes during each session and, immediately upon conclusion, completed a descriptive rating across six predefined dimensions: Engagement, Interaction, Emotional Expression, Focus, Creativity, and Stress. As observations were conducted by a single researcher without inter-rater reliability procedures, these records constitute descriptive process data complementing quantitative findings rather than inferential statistical evidence (Sandelowski, 2001). Ratings were visualized using heatmaps and radar charts; the complete observation framework is provided in Supplementary Materials S1. For the radar charts, each axis value represents the mean of the two sessions within the corresponding week (e.g., Week 1 = mean of Sessions 1–2), and stress ratings were reverse-coded as stress relief (5 minus the raw stress rating) so that all six dimensions could be displayed on a consistent higher-is-more-positive scale.

#### 2.5.2. Six-Month Follow-Up Interviews

Individual semi-structured follow-up interviews were conducted with all eight experimental group participants (P1–P8) on 19 July 2025, each lasting approximately 30 min in Mandarin Chinese. With informed consent, interviews were audio-recorded and transcribed verbatim (1033–2010 words per participant). Questions addressed changes in emotional regulation, coping strategy transfer, and sustained creative practice; participants were not asked to recount DV experiences to minimize re-traumatization risk. The guide was reviewed by the mental health counsellor (E2) for psychological safety. The interview guide is provided in Supplementary Materials S2. All transcripts were imported into NVivo 15.

### 2.6. Statistical Analysis

Internal consistency reliability (Table 2) was assessed using IBM SPSS Statistics Version 20 (IBM Corp., 2011). All inferential analyses—including baseline group equivalence (Table 3), within-group comparisons (Table 4), and between-group change-score and follow-up comparisons (Table 5, Table 6 and Table 7)—were conducted in R Version 4.5.0 (R Core Team, 2025).

Within-group longitudinal changes (T0 to T1; T1 to T2; T0 to T2) were assessed with the Wilcoxon signed-rank test. Baseline group equivalence and between-group absolute score comparisons at T2 used the Mann–Whitney U test. Short-term (Δ = T1 − T0) and six-month (Δ = T2 − T0) change score comparisons between groups also used the Mann–Whitney U test to directly compare magnitudes of change (Conover, 1999). Wilcoxon and Mann–Whitney tests were computed using the normal approximation with continuity correction. Effect sizes were calculated as r = |Z|/√N, where N represents the total number of observations (N = 16), applied consistently across all tests to ensure comparability (Field, 2018; Rosenthal, 1991), and interpreted per Cohen (1988): 0.10 = small, 0.30 = medium, 0.50 = large. Z values are reported as absolute values throughout. Hodges–Lehmann 95% confidence intervals (CIs) were computed in R using wilcox.test(conf.int = TRUE) and are reported for the primary between-group change-score comparisons in Table 5 and Table 6 (Hodges & Lehmann, 1963). All tests were two-tailed; α = 0.05.

### 2.7. Qualitative Analysis

Interview data were analyzed using Braun and Clarke’s (2006, 2019) reflexive thematic analysis, following their original six-phase framework, conducted on original Chinese transcripts; English translations served reporting purposes only. All procedures were completed in NVivo 15. Trustworthiness followed Lincoln and Guba (1985): credibility through peer debriefing with the corresponding author; confirmability through an NVivo audit trail; and reflexivity through explicit acknowledgement of the researcher’s single-observer role as a potential source of observational bias, discussed further in Section 4, as well as the researcher’s active, interpretive role in generating themes from the interview data rather than passively identifying pre-existing patterns.

### 2.8. Ethics

This study was approved by the Human Research Ethics Committee of Universiti Sains Malaysia (JEPeM-USM; protocol code: USM/JEPeM/PP/24040359) and conducted in accordance with the Declaration of Helsinki (World Medical Association, 2013). Written informed consent was obtained on-site from all participants prior to data collection; participation was voluntary and withdrawal at any stage carried no consequences. Data were anonymized (experimental group: P1–P8; control group: P9–P16) and securely stored; original paper documents will be destroyed upon study completion per institutional requirements. Control group and reserve pool members were offered voluntary arts-based workshop access upon completion of data collection (American Psychological Association, 2017). No adverse psychological events requiring psychiatric intervention were recorded throughout this study.

## 3. Results

### 3.1. Participant Characteristics and Baseline Equivalence

All 16 participants completed assessments at baseline (T0), post-intervention (T1), and six-month follow-up (T2), yielding a retention rate of 100%. As shown in Table 8, the sample comprised predominantly middle-aged and older women, with 81.3% aged 40 years or above and 37.5% aged 60 or older. Educational attainment was low, with 75% having completed junior secondary schooling or below; socioeconomic status was low in 62.5% of participants and moderate in the remaining 37.5%; and 56.3% had been married for 30 or more years. All participants (100%) reported a history of psychological violence, and 87.5% reported physical violence.

Internal consistency reliability of the three instruments across all assessment points was acceptable to good (Cronbach’s α = 0.73–0.85; Table 2).

Pre-intervention comparisons confirmed initial group equivalence across all outcome measures (Table 3): SDS (U = 44.00, *p* = 0.227), SAS (U = 35.50, *p* = 0.753), and RSES (U = 39.00, *p* = 0.490). No statistically significant baseline differences were detected between the experimental and control groups.

### 3.2. Short-Term Intervention Outcomes

#### 3.2.1. Within-Group Changes

**Figure 3:**
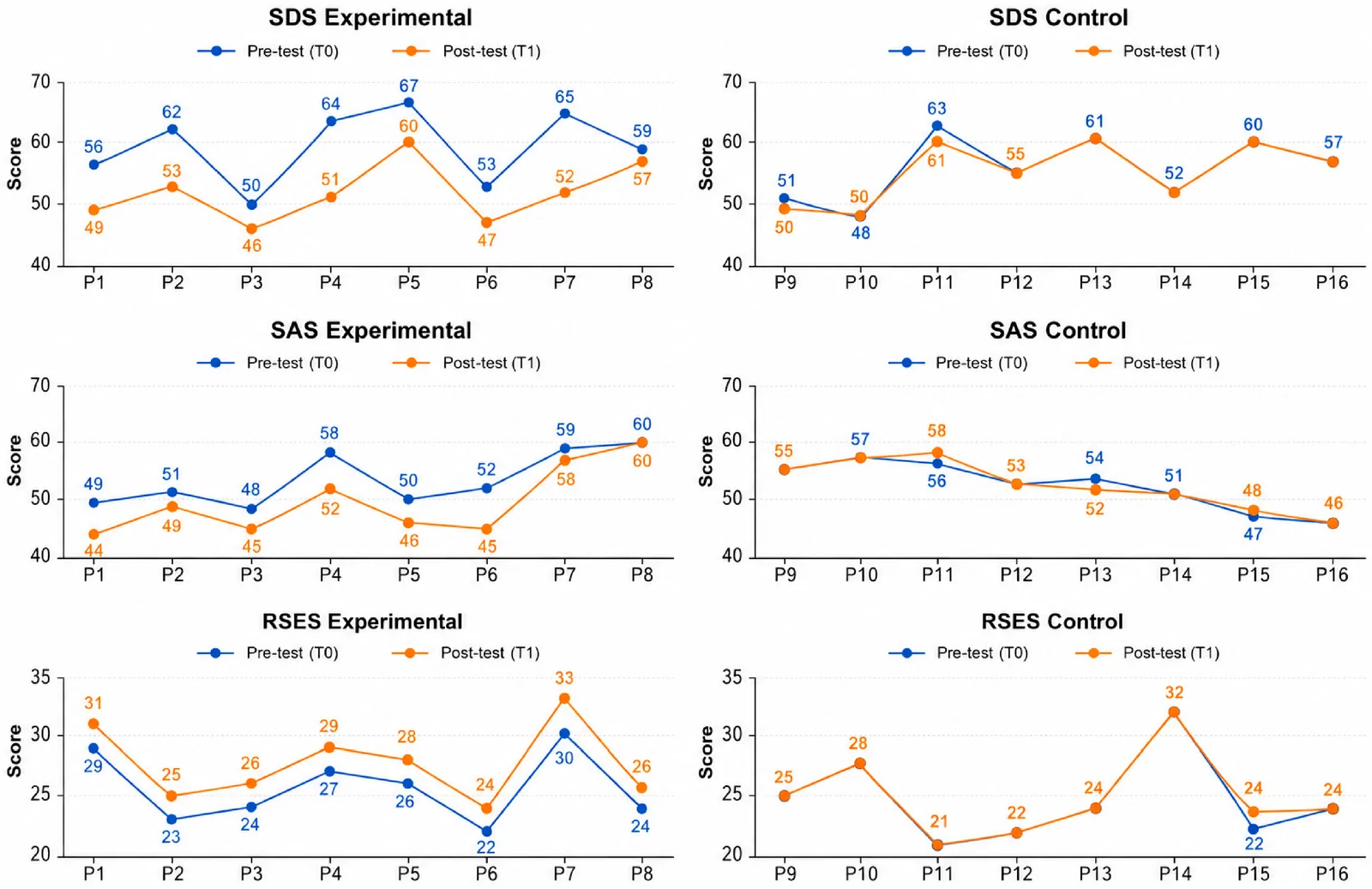
Individual trajectories of SDS, SAS, and RSES scores from baseline (T0) to post-intervention (T1) for experimental and control groups.

At T1, the experimental group demonstrated statistically significant improvements across all three outcome measures (Table 4; Figure 3). As shown in Figure 3, these improvements were evident across nearly all individual trajectories in the experimental group, in contrast to the flat trajectories observed in the control group, suggesting that the observed short-term benefits were not driven by a small number of high responders. Depression scores declined from 59.50 (SD = 6.07) to 51.88 (SD = 4.79; Z = 2.46, *p* = 0.014, r = 0.61); anxiety scores declined from 53.38 (SD = 4.84) to 49.88 (SD = 6.22; Z = 2.28, *p* = 0.022, r = 0.57); and self-esteem increased from 25.62 (SD = 2.88) to 27.75 (SD = 3.11; Z = 2.64, *p* = 0.008, r = 0.66). All three effect sizes were large (r = 0.57–0.66). Post-intervention, the experimental group means for SDS (51.88) and SAS (49.88) fell below the respective Chinese clinical cut-off scores (SDS ≥ 53; SAS ≥ 50), whereas control group means remained above both thresholds throughout. The control group showed no change on any measure over the same period (Z = 0.00, *p* = 1.000 for all three measures).

#### 3.2.2. Between-Group Change Score Comparisons

Between-group comparisons of short-term change scores (Δ = T1 − T0) yielded large, statistically significant differences across all three measures (Table 5). The experimental group demonstrated substantially greater reductions in depression than the control group (Δ: −7.62 vs. −0.12; U = 0.50, Z = 3.31, *p* < 0.001, r = 0.83, 95% CI [−12.00, −4.00]). Comparable patterns were observed for anxiety (Δ: −3.50 vs. 0.12; U = 5.00, Z = 2.86, *p* = 0.004, r = 0.72, 95% CI [−6.00, −1.00]) and self-esteem (Δ: 2.12 vs. 0.25; U = 60.50, Z = 3.30, *p* = 0.001, r = 0.83, 95% CI [2.00, 2.00]). Effect sizes ranged from r = 0.72 to r = 0.83, and all confidence intervals excluded zero. Cross-sectional comparisons at T1 did not reach conventional significance for depression (*p* = 0.126) or anxiety (*p* = 0.269), with self-esteem approaching significance (*p* = 0.063).

### 3.3. Behavioral Observations

**Figure 4:**
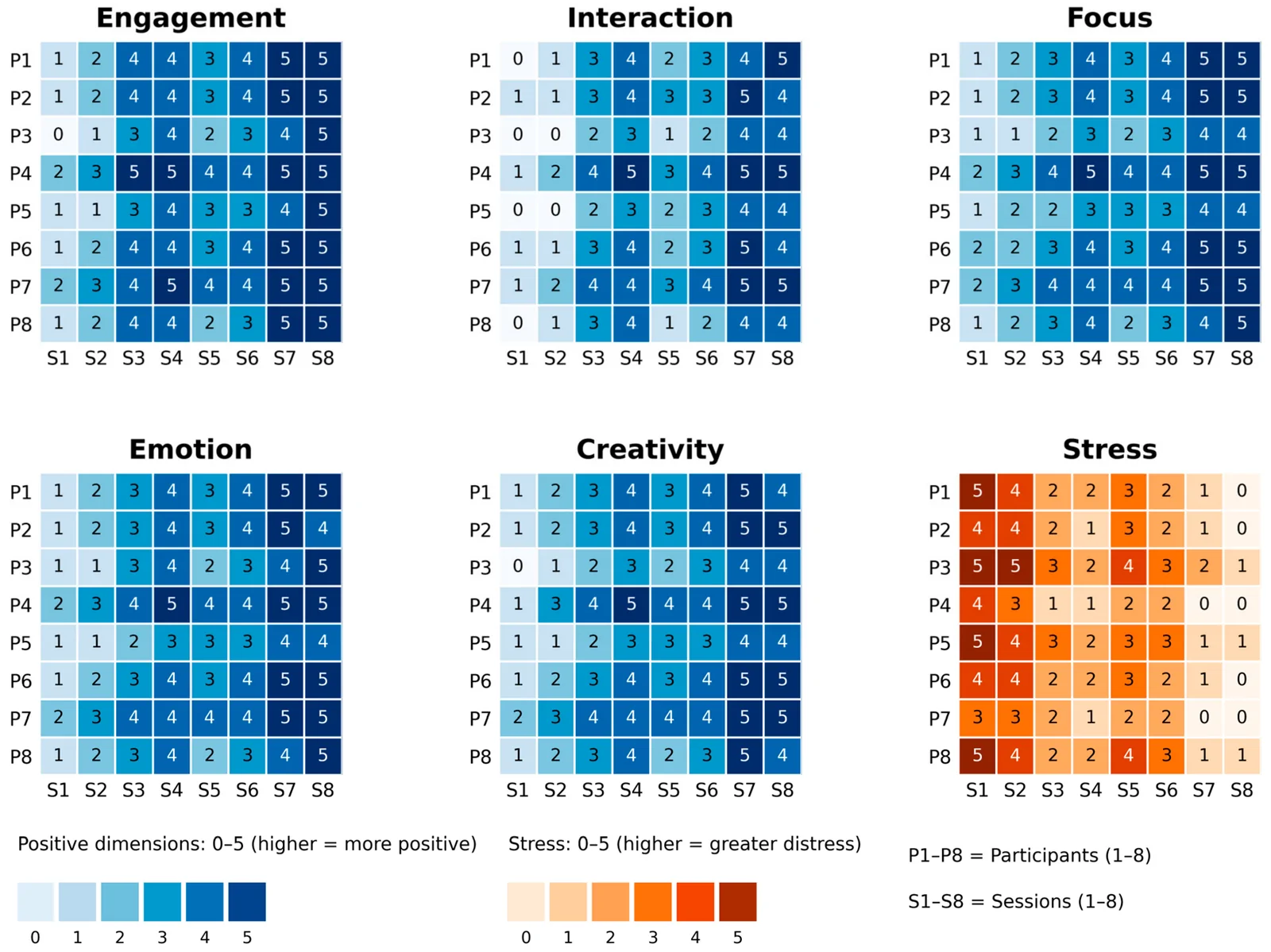
Heatmap of descriptive behavioral ratings across six dimensions for all eight sessions.

Structured observation records served as descriptive process data documenting behavioral trajectories across the eight sessions. To visualize these patterns, session-by-session ratings across all eight sessions and six dimensions were displayed as a heatmap (Figure 4), enabling direct comparison of trajectories both within and across participants, while individual-level radar profiles were used to examine whether observed gains were consistent across participants rather than concentrated in a subset of the sample.

#### 3.3.1. Early Sessions (S1–S2)

Initial sessions were marked by pervasive somatic stress responses and minimal participation. P1 “sat nearest to the door with clenched hands,” scanning the room throughout; P8 displayed “shallow breathing and rigid posture, unable to relax her shoulders even when seated”; and P5 was observed “picking up materials and putting them down repeatedly.” P3 remained in the corner and declined to present her mask to the group, whispering that it was “not finished.” The heatmap (Figure 4) illustrates that stress ratings were consistently elevated across participants in S1–S2, while engagement, interaction, and creativity ratings remained low.

#### 3.3.2. Middle Sessions (S3–S5)

**Figure 5:**
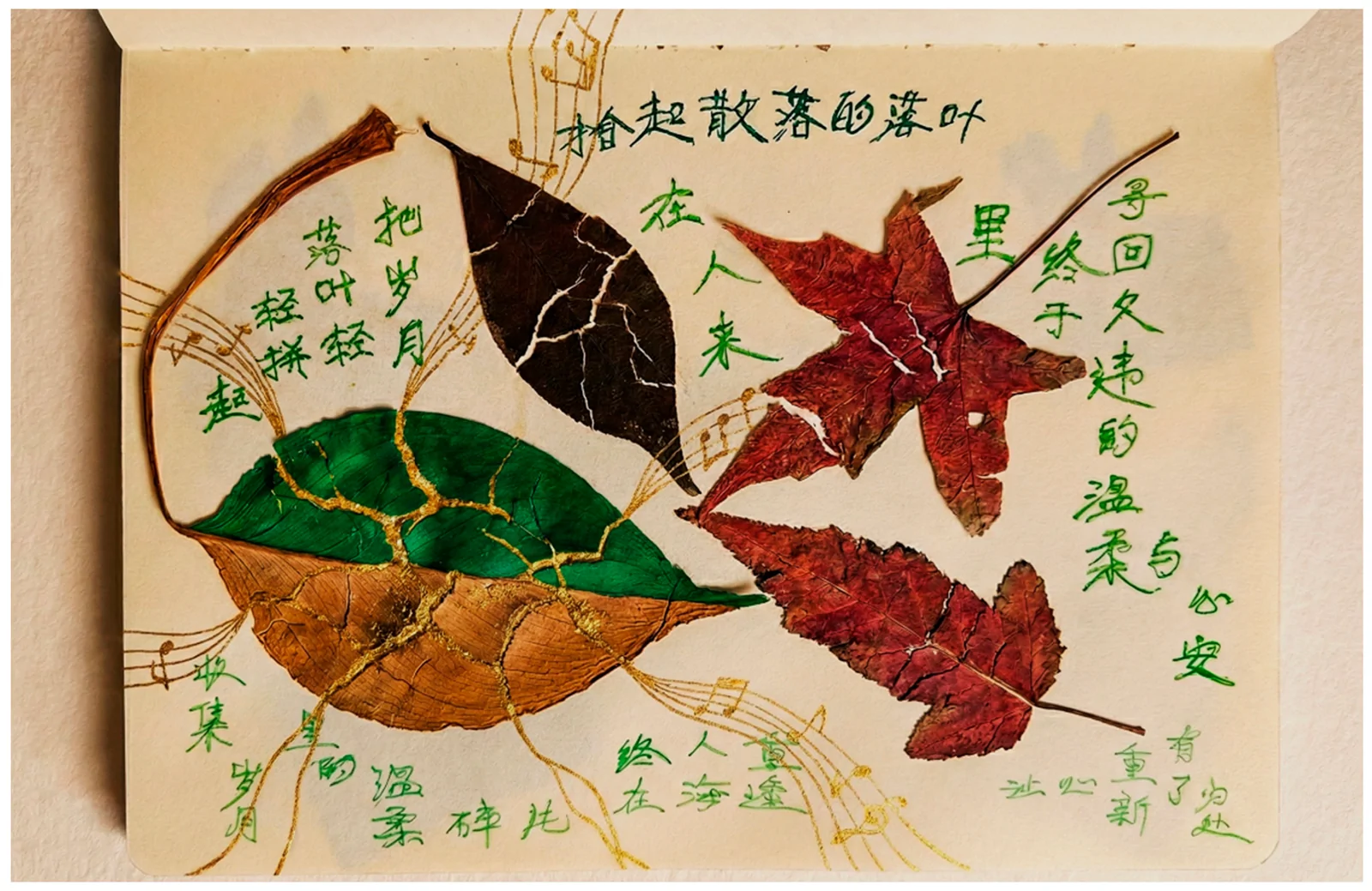
Photograph of P1’s reconstructed collage artwork created during Session 5.

The introduction of the sound module (S3) and collage activity (S5) marked a discernible shift in behavioral patterns. During S3, P8—previously rigid—was observed “swaying her body slightly with the rhythm.” In S5, P1 reassembled torn paper fragments into a unified image (Figure 5); observation notes recorded that “her breathing slowed and her movements became fluid,” and she spontaneously remarked, “I didn’t know I could fix it.” Within the same session, P3 completed an independent collage for the first time, supported by the group’s non-judgmental atmosphere. As illustrated in Figure 4, stress ratings began declining from S3, with concurrent upward trends in engagement and creativity. Focus and emotion ratings followed a similar upward trajectory, though both dipped slightly at S5 before recovering by S6, coinciding with the more emotionally demanding image-deconstruction activity, a pattern consistent with the expected intensification of affective engagement during trauma-processing tasks rather than disengagement.

#### 3.3.3. Late Sessions (S6–S8)

**Figure 6:**
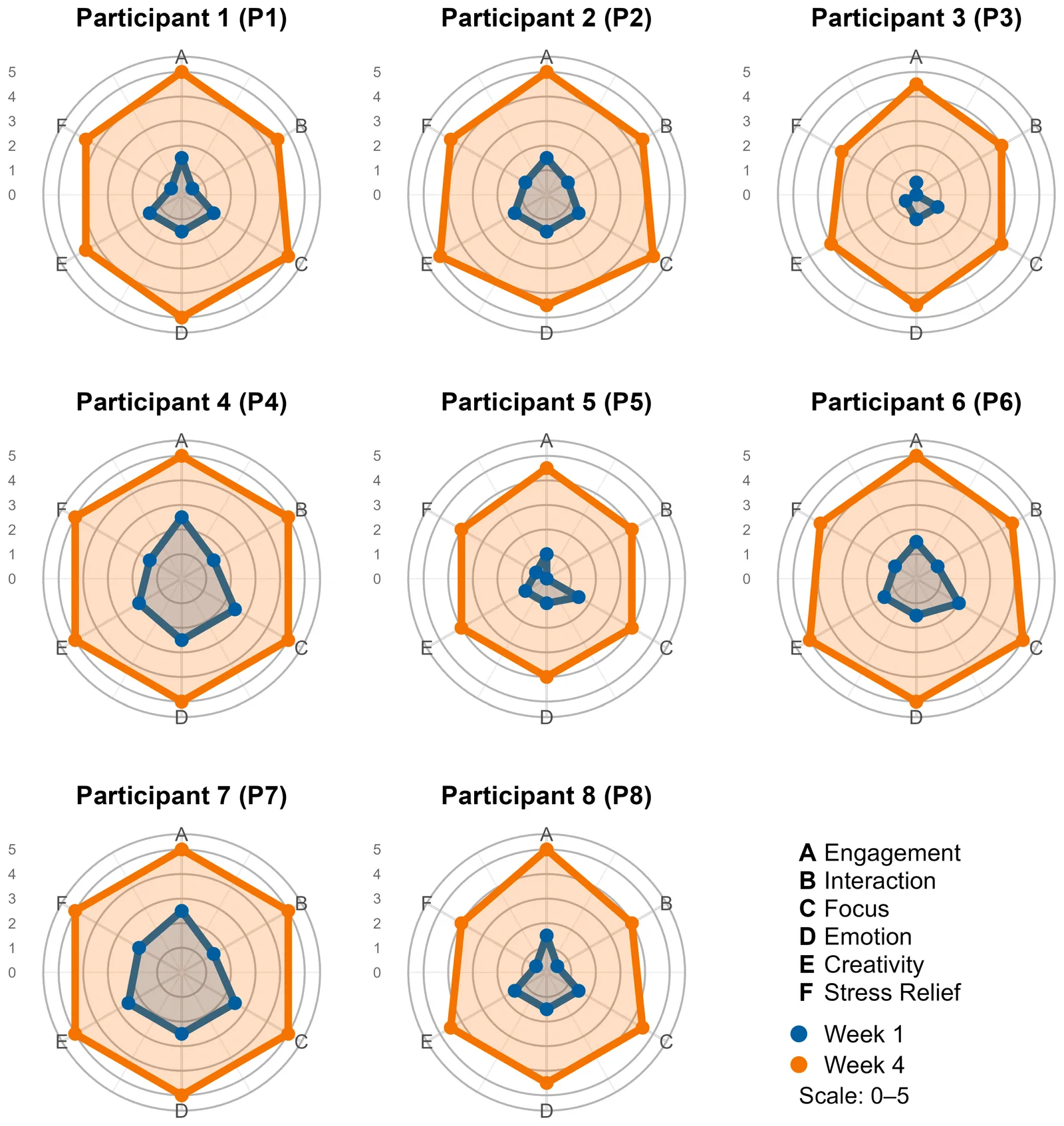
Radar charts comparing behavioral ratings at Week 1 and Week 4 for all eight experimental group participants.

By the final phase, interaction and creativity ratings had reached their highest levels across the cohort. P4 and P7 were observed “actively assisting peers” and “initiating group sharing,” while P2 “confidently displayed a complete portfolio.” In the closing session, P8 “initiated a hug with every member.” The radar charts (Figure 6) show that all eight participants exhibited outward expansion across the six behavioral dimensions by Week 4, with P3 displaying one of the most pronounced visual changes. This pattern indicates that gains were broadly distributed across the cohort rather than concentrated in a subset of participants, an important consideration given the small sample size of this pilot study.

The session-by-session behavioral trajectories described above were consistent in direction with the quantitative improvements reported in Section 3.2.

### 3.4. Six-Month Follow-Up: Quantitative Outcomes

#### 3.4.1. Stability of Intervention Effects

At T2, no statistically significant regression from post-intervention levels was observed in the experimental group: SDS (Z = 0.352, *p* = 0.725), SAS (Z = 0.679, *p* = 0.497), RSES (Z = 0.213, *p* = 0.832). Improvements relative to baseline remained statistically significant for all three measures (SDS: *p* = 0.012; SAS: *p* = 0.018; RSES: *p* = 0.027), confirming that gains achieved at T1 were sustained through the six-month follow-up. The control group showed no significant change over the same interval on any measure (all *p* > 0.16).

#### 3.4.2. Six-Month Change Score Comparisons

Between-group comparisons of six-month change scores (Δ = T2 − T0) yielded significant group differences across all three measures (Table 6). Relative to the control group, the experimental group showed markedly greater reductions in depression (Δ: −8.50 vs. −0.88; U = 2.00, Z = 3.11, *p* = 0.002, r = 0.78, 95% CI [−13.00, −4.00]) and anxiety (Δ: −5.00 vs. 0.50; U = 6.00, Z = 2.69, *p* = 0.007, r = 0.67, 95% CI [−9.00, −2.00]), alongside greater gains in self-esteem (Δ: 2.00 vs. 0.00; U = 53.00, Z = 2.19, *p* = 0.029, r = 0.55, 95% CI [0.00, 4.00]). Effect sizes remained large across all indicators (r = 0.55–0.78).

#### 3.4.3. Between-Group Comparisons at T2

**Figure 7:**
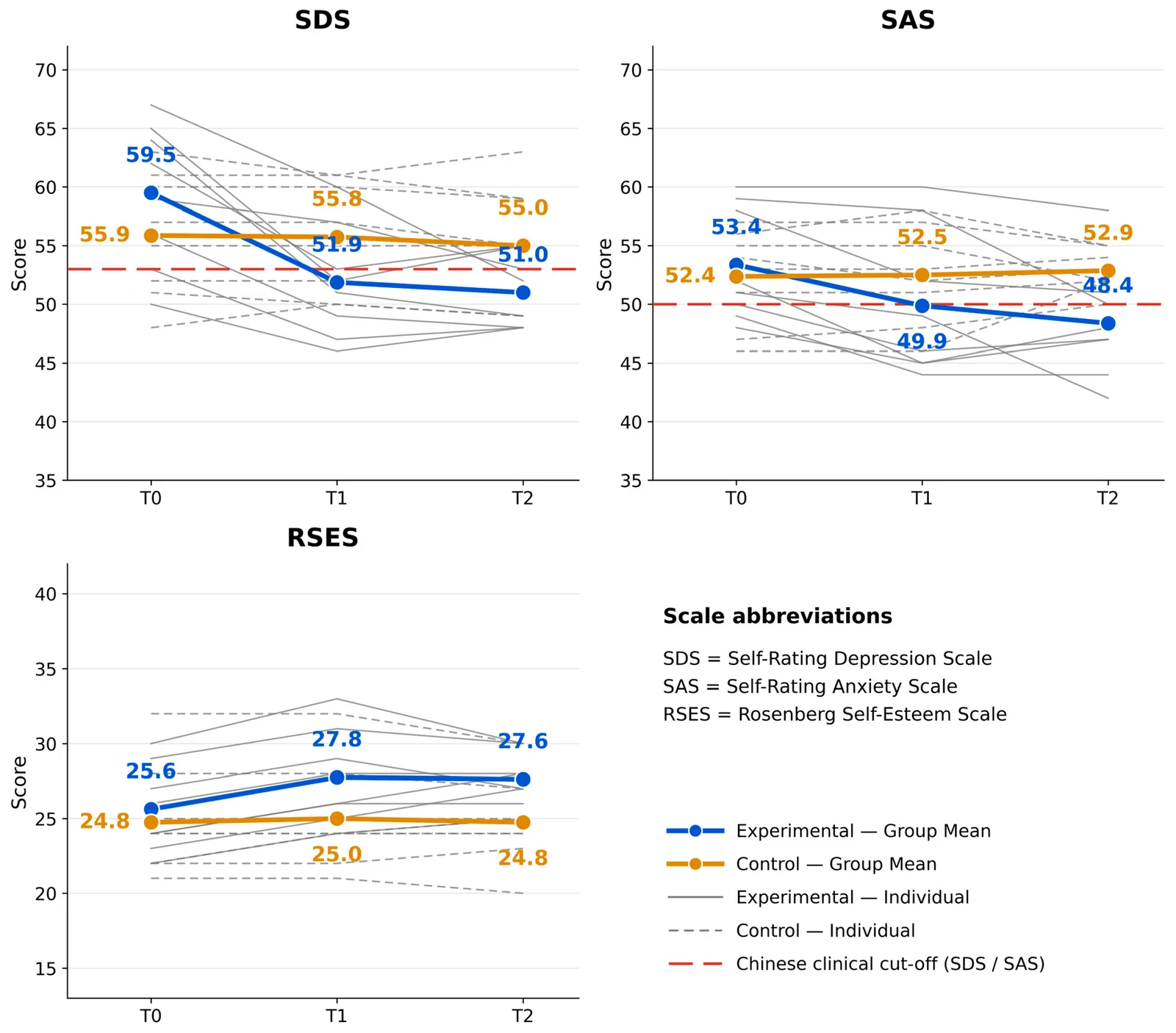
Longitudinal trajectories of SDS, SAS, and RSES scores from T0 to T2, with Chinese clinical cut-off thresholds indicated.

Absolute score comparisons at T2 confirmed significant group differences for anxiety (SAS: U = 9.50, *p* = 0.020, r = 0.58) and self-esteem (RSES: U = 53.00, *p* = 0.030, r = 0.54), with depression approaching significance (SDS: U = 15.00, *p* = 0.079, r = 0.44; Table 7; Figure 7). As shown in Figure 7, these between-group differences were sustained across the entire follow-up window, with the experimental group’s trajectory remaining below the Chinese clinical cut-offs for both SDS and SAS from post-intervention through six-month follow-up, while the control group’s trajectory remained above threshold throughout. At T2, experimental group means for SDS (51.00) and SAS (48.38) remained below the respective Chinese clinical thresholds (≥53 and ≥50), whereas control group means for both indicators (SDS: 55.00; SAS: 52.88) remained above both cut-offs. Substantively, this pattern suggests that the intervention group moved from clinically elevated depression/anxiety toward below-threshold mean scores, whereas the control group showed little comparable recovery.

### 3.5. Six-Month Follow-Up: Qualitative Findings

Inductive thematic analysis of follow-up interview data from all eight experimental group participants (P1–P8) generated five themes (Table 9). Three themes directly addressed sustained psychological outcomes and are presented in detail below. The remaining two—participant reflections on specific intervention activities and reported challenges—are briefly noted, as they relate primarily to intervention delivery rather than to outcome sustainability.

#### 3.5.1. Affective and Emotional Change

Participants described stable improvements in emotional functioning in everyday life. One participant contrasted her current morning routine with her pre-intervention state: “Now when I get up in the morning, I don’t lie there not wanting to move anymore. I feel like there is a little bit of colour in the day” (P1). Another described drawing on sensory memories from the sound session as an ongoing strategy for managing anxiety: “Now, when I feel panicked at home, I try to remember that sound, that vibration. It helps me breathe again” (P3).

#### 3.5.2. Cognitive and Self-Perception Reconstruction

Participants reported enduring changes in self-concept and perceived capability. One participant articulated a fundamental shift in identity: “Before I thought I was just a victim, a failure. Now I think I’m a survivor… that itself is a kind of strength” (P6). Another described a pivotal moment of self-expression that continued to shape her sense of self: “I stood up and said, ‘I found my own voice.’ After saying it I cried… At that moment I felt, I can finally be myself” (P8). A third offered concrete behavioral evidence of sustained self-efficacy: “I have confidence now, dare to make decisions. I changed jobs last month—before I wouldn’t dare think of it” (P4).

#### 3.5.3. Behavioral and Social Manifestations

**Figure 8:**
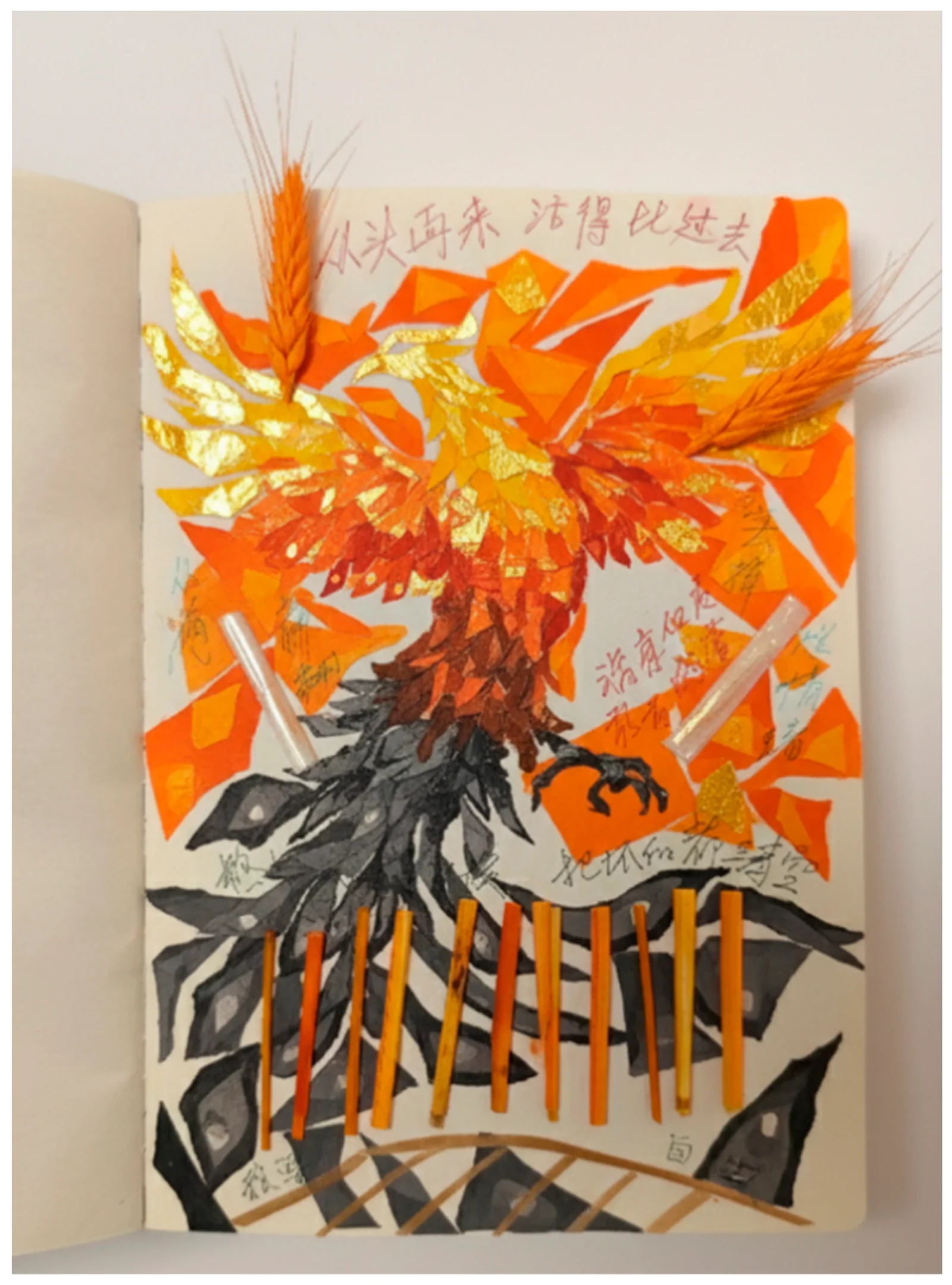
Photograph of P4’s visual journal entry maintained during the six-month follow-up period.

Participants described lasting changes in coping and social engagement. One participant had integrated creative practice into her approach to interpersonal conflict: “I went home and painted for a while; after painting my heart felt much better, and then I went to talk properly with my neighbour” (P1). Another described a sustained move from social withdrawal to active community participation: “I even organized a small book club—exchanging ideas with friends. These things were impossible before” (P7). P4 continued maintaining a visual journal throughout the follow-up period (Figure 8).

#### 3.5.4. Additional Themes

Participants also reflected on specific intervention activities that continued to support coping after the program ended; the sound resonance activity was the most frequently cited. Alongside these positive reflections, some participants described initial reluctance to engage an emotional discomfort during certain activities.

### 3.6. Mixed-Methods Integration

Findings across the three data sources showed a consistent pattern of change. The significant between-group difference in anxiety at T2 (U = 9.50, *p* = 0.020) aligned with P3’s account of using sound memories as an ongoing coping strategy. The sustained group difference in self-esteem (U = 53.00, *p* = 0.030) corresponded with P6’s narrative of identity reconstruction and P4’s reported career decision. The session-by-session behavioral trajectories—declining stress ratings alongside rising engagement and creativity—were consistent in direction with the large between-group effect sizes observed in short-term change scores (r = 0.72–0.83).

Overall, findings from quantitative outcomes, behavioral observations, and participant narratives showed a consistent pattern, providing complementary evidence in support of the preliminary findings of this pilot study (Figure 3, Figure 4, Figure 5, Figure 6, Figure 7 and Figure 8).

## 4. Discussion

This pilot study provides preliminary evidence that a culturally adapted, community-based arts intervention may support both short-term psychological improvement and sustained recovery outcomes across a six-month follow-up period among women survivors of DV in mainland China, a population for whom structured psychosocial interventions remain critically underexplored. The following sections discuss these findings in relation to the existing literature, reflect on the methodological contribution of the mixed-methods design, and consider the implications for future research and practice.

### 4.1. Short-Term Intervention Effects

One of the principal short-term findings was the simultaneous improvement across all three psychological outcome measures, each accompanied by a large effect size, a pattern that is not consistently observed in the existing arts-based intervention literature with DV survivors.

Two limitations characterize much of the prior work in this area: the absence of a concurrent control group, which makes it difficult to distinguish intervention effects from spontaneous recovery; and inconsistent effect size reporting, which constrains meaningful cross-study comparison. Aktaş Özkafacı and Eren (2020) and Teague et al. (2006) both reported statistically significant improvements, but neither study included a control group; Sabina et al. (2023), in a controlled design with 87 participants, found a partial η^2^ of 0.05 for self-esteem, a small-to-medium effect. Against this backdrop, the between-group change score effect sizes observed in the present study (r = 0.72–0.83), alongside within-group effect sizes of r = 0.57–0.66, represent a stronger preliminary signal, though direct comparisons remain limited by differences in intervention content, outcome measures, and analytic approaches.

These findings suggest that the sequential modular structure of Canvas of Strength—progressing from self-exploration and emotional regulation through cognitive reconstruction to ongoing creative integration—may constitute a mutually reinforcing therapeutic sequence that amplifies short-term outcomes. Self-esteem yielded the largest effect size and strongest statistical significance of the three indicators (r = 0.66, *p* = 0.008), in contrast to studies such as Teague et al. (2006) in which self-esteem did not reach significance. This may reflect the deliberate inclusion of identity reconstruction activities within the intervention design, though this interpretation warrants formal testing in subsequent research.

Although clinical remission cannot be inferred from a pilot sample of this size, the observation that post-intervention experimental group means for SDS (51.88) and SAS (49.88) both fell below the respective Chinese clinical cut-off scores while control group means remained above both thresholds suggests that the magnitude of improvement may carry clinical as well as statistical relevance.

### 4.2. Six-Month Follow-Up: The Core Contribution of This Study

The principal methodological contribution of this study is the provision of six-month follow-up data within a controlled design—a feature that remains notably absent from most comparable arts-based intervention studies with DV survivors, and one that strengthens the evidentiary basis of the present findings relative to much of the prior literature.

The absence of follow-up data has emerged as a near-systematic gap in this field. Vela et al. (2016) and Ikonomopoulos et al. (2017) both explicitly identified this as a study limitation; Aktaş Özkafacı and Eren (2020) similarly did not include a follow-up assessment. Giesbrecht et al. (2022) provide a valuable reference in their longitudinal study of Indigenous Canadian DV survivors (N = 101), which documented sustained reductions in depression and anxiety at a one-year follow-up, though without a control group. The present study, within a controlled framework, found no statistically significant regression from post-intervention gains at T2 across any of the three outcome measures; between-group change score comparisons remained significant across all indicators (r = 0.55–0.78), and between-group absolute score comparisons at T2 reached significance for anxiety (*p* = 0.020) and self-esteem (*p* = 0.030). These patterns provide preliminary support for the durability of intervention effects over a six-month period.

One plausible explanation for this sustained improvement is that participants continued to integrate the creative practices learned during the program into their everyday coping repertoires after the intervention ended. This interpretation is consistent with participant narratives: P3 described drawing on sensory memories from the sound resonance sessions as an ongoing anchor for anxiety regulation; P4 maintained a visual journal throughout the follow-up period; and P1 incorporated painting into her approach to interpersonal conflict management. Skop et al. (2022) and Allen and Wozniak (2011) similarly identified creative empowerment and self-reconstruction as central dimensions of recovery in DV survivors, and the present qualitative findings are directionally consistent with these frameworks. That said, this interpretation remains exploratory; the present study was not designed to formally test intervention mechanisms or mediating pathways, and these questions require dedicated investigation in future research.

The significance of these findings is further amplified by the Chinese mainland research context. Yuan and Hesketh (2021) documented depression prevalence rates of 65.8% to 75.8% among Chinese women experiencing different forms of IPV, with IPV associated with a more than twofold increase in the odds of depression. Yet systematic and scoping reviews by Zhao et al. (2022) and Li et al. (2020) confirm that intervention research targeting DV survivors in mainland China remains sparse, with existing studies concentrated almost exclusively on epidemiology and risk factors. While Xu et al. (2025) demonstrate that arts-based approaches have been applied with Chinese clinical populations more broadly, to our knowledge, no published study has evaluated a structured arts-based intervention for DV survivors in mainland China and reported follow-up data. The present study therefore offers a preliminary empirical reference point for this underserved area of inquiry.

### 4.3. Methodological Contribution of the Mixed-Methods Design

The value of the mixed-methods design adopted in this study lies not simply in the triangulation of data sources, but in its capacity to illuminate how recovery unfolded across the intervention, rather than merely whether it occurred. This distinction carries substantive implications for DV survivor intervention research.

Quantitative outcome data captured the magnitude of change but cannot account for the processes through which change accumulated. Behavioral observation across the eight sessions revealed a three-phase trajectory—from early freeze responses characterized by elevated stress and minimal engagement, through a non-verbal breakthrough phase in which creative media functioned as a mediating channel, to a final phase marked by emerging agency and social connection—providing a process-level account that outcome data alone could not supply. Qualitative interview data then offered participants’ own descriptions of how these shifts extended into daily life across the six-month follow-up. Across the three data sources, a consistent directional pattern emerged, providing complementary support for the preliminary conclusions of this pilot study.

This design orientation carries broader implications for the field. DV survivor intervention research that relies exclusively on standardized outcome measurement risks missing critical within-intervention dynamics: behavioral changes often precede detectable shifts in scale scores, and qualitative data reveal the processes of meaning-making that underlie statistical change. Mixed-methods designs may offer a more complete account of how psychological recovery unfolds across cognitive, behavioral, and social dimensions, information that is potentially as important for intervention refinement as aggregate outcome data.

### 4.4. Limitations

Several limitations should be considered when interpreting these preliminary findings. The most consequential constraint on internal validity is sample size. With eight participants per group and a total analytic sample of N = 16, this study is appropriately scaled to its pilot purpose of generating preliminary effect size estimates and feasibility data (Thabane et al., 2010), but cannot support definitive statistical inference. Despite the large effect sizes observed, the influence of Type II error and chance variation cannot be excluded in samples of this magnitude; the trend-level between-group difference for depression at T2 (*p* = 0.079) may itself reflect insufficient statistical power rather than the absence of a true effect.

Behavioral observation also introduced an important source of potential bias. Behavioral records were completed by a single researcher throughout all eight sessions without inter-rater reliability procedures, and the researcher simultaneously performed logistical support functions during the intervention. This dual role represents a structural limitation on the objectivity of the observational data; while the use of immediate post-session descriptive ratings based on contemporaneous field notes was intended to reduce retrospective distortion, the absence of a second independent observer cannot be fully compensated for by procedural measures alone.

Furthermore, the open-label nature of the intervention inherently meant that neither participants nor intervention team members were blinded to group allocation. This leaves open the possibility that expectancy effects or social desirability responses contributed to the observed outcomes, a limitation inherent to group intervention research of this nature.

Additional constraints merit consideration. Recruitment from a community administrative mediation registry may have introduced systematic sampling bias, as women who have engaged with formal dispute resolution may differ from the broader DV survivor population in help-seeking orientation and violence exposure profile. The single-site design limits geographic and cultural generalizability. The six-month follow-up represents a medium-term observation window, and whether the effects persist beyond this period remains exploratory. Finally, qualitative interviews were conducted exclusively with experimental group participants, precluding comparison with control group experiential trajectories and thereby constraining the depth of the mixed-methods integration.

### 4.5. Implications and Future Directions

The practical significance of these preliminary findings centers on a question that extends beyond the efficacy of any single program: whether arts-based approaches can serve as a structurally viable modality for reaching DV survivors in low-resource community settings, and whether they can do so in ways that reduce the barriers that have historically limited this population’s engagement with conventional mental health services.

Several structural features of arts-based delivery are relevant here. Framing the intervention as a creative workshop rather than clinical psychotherapy substantially lowers the threshold of help-seeking stigma, allowing participation without the self-identification as a mental health service user that formal counselling typically requires (Malchiodi, 2011). Arts-based activities also provide a non-verbal channel for emotional expression, a feature of particular relevance for trauma survivors for whom verbal disclosure of abuse carries a risk of retraumatization. The low material cost of the activities used in Canvas of Strength addresses the resource constraints of community settings without specialist clinical infrastructure.

The modular structure of the intervention may, furthermore, facilitate future implementation by trained community social workers, potentially extending the program’s reach beyond the capacity of specialist staffing. Such characteristics align closely with task-sharing and community-based mental health strategies advocated for settings with limited specialist resources. However, whether social workers can deliver the full protocol independently, and with what level of training, requires formal evaluation before such claims can be substantiated. In mainland China, where professional mental health resources in community settings remain critically limited (Li et al., 2020), these features collectively suggest a potential pathway toward more accessible DV survivor support.

Future research should address the following priorities: determining sample sizes adequate for powered hypothesis testing; incorporating an active control condition to isolate the specific contribution of arts-based activities from non-specific group effects; conducting multi-site replication to assess generalizability; formally measuring candidate mediating variables—including emotional regulation capacity and self-efficacy—to test the recovery pathways suggested by the qualitative data; and extending the follow-up period to twelve months or beyond.

Collectively, these findings should be interpreted as preliminary rather than definitive. They do, however, provide an empirical foundation for larger-scale trials of culturally adapted, community-based arts interventions for women survivors of DV in mainland China, and suggest that such interventions may offer a viable and structurally accessible route to psychological support for a population whose needs have, to date, received insufficient research attention.

## 5. Conclusions

This mixed-methods pilot study provides preliminary empirical evidence supporting the potential value of a culturally adapted community-based arts intervention for women survivors of DV in mainland China, where structured psychosocial intervention research remains limited. Quantitative findings suggested improvements in depression, anxiety, and self-esteem that were largely maintained over a six-month follow-up. Behavioral observations documented progressive changes in participants’ engagement and sense of agency throughout the intervention, while qualitative interviews indicated that these changes continued to influence participants’ everyday coping and recovery beyond the intervention period. Together, these complementary findings provide preliminary support for a sustained pattern of psychological recovery across quantitative, behavioral, and qualitative data.

These findings should be interpreted in light of this study’s pilot nature, including the small sample size, single-site design, and open-label implementation. Nevertheless, this study establishes an initial empirical foundation for culturally adapted community-based arts interventions for women survivors of DV in mainland China and provides an important reference for future adequately powered randomized controlled trials examining intervention effectiveness, mechanisms of change, and implementation in community settings, contributing to an emerging evidence base for psychosocial intervention with women survivors of DV in mainland China.

## Figures and Tables

**Table 2:** Internal consistency reliability (Cronbach’s α) of psychological measures across time points.

Measure	Number of Items	Pre-Test	Post-Test	Follow-Up
Depression (SDS)	20	0.81	0.82	0.82
Anxiety (SAS)	20	0.82	0.85	0.76
Self-Esteem (RSES)	10	0.73	0.73	0.75

Note. SDS = Zung Self-Rating Depression Scale; SAS = Zung Self-Rating Anxiety Scale; RSES = Rosenberg Self-Esteem Scale. Cronbach’s α values ≥ 0.70 indicate acceptable internal consistency, and values ≥ 0.80 indicate good internal consistency. Analyses were conducted using IBM SPSS Statistics Version 20.

**Table 3:** Baseline group equivalence on psychological measures (T0).

Measure	Experimental M (SD)	Control M (SD)	U	*p*
Depression (SDS)	59.50 (6.07)	55.88 (5.30)	44.00	0.227
Anxiety (SAS)	53.38 (4.84)	52.38 (4.07)	35.50	0.753
Self-Esteem (RSES)	25.62 (2.88)	24.75 (3.65)	39.00	0.490

Note. Between-group baseline differences were assessed using the Mann–Whitney U test (N = 16). SDS = Zung Self-Rating Depression Scale (Chinese clinical cut-off: ≥53; Wang & Wang, 1999); SAS = Zung Self-Rating Anxiety Scale (Chinese clinical cut-off: ≥50; Y. Liang et al., 2020); RSES = Rosenberg Self-Esteem Scale. All *p* > 0.20, indicating no statistically significant baseline differences between groups. All analyses were conducted in R (Version 4.5.0), applying normal approximation with continuity correction.

**Table 4:** Short-term intervention effects: within-group pre-test to post-test comparisons.

Measure	Group	Pre-Test M (SD)	Post-Test M (SD)	Z	*p*	r
SDS	Experimental	59.50 (6.07)	51.88 (4.79)	2.46	0.014 *	0.61
	Control	55.88 (5.30)	55.75 (4.71)	0.00	1.000	0.00
SAS	Experimental	53.38 (4.84)	49.88 (6.22)	2.28	0.022 *	0.57
	Control	52.38 (4.07)	52.50 (4.17)	0.00	1.000	0.00
RSES	Experimental	25.62 (2.88)	27.75 (3.11)	2.64	0.008 **	0.66
	Control	24.75 (3.65)	25.00 (3.51)	0.00	1.000	0.00

Note. Within-group changes (T0 to T1) were assessed using the Wilcoxon signed-rank test (n = 8 per group). Effect size r = |Z|/√N (N = 16), interpreted per Cohen (1988): 0.10 = small, 0.30 = medium, 0.50 = large. SDS = Self-Rating Depression Scale; SAS = Self-Rating Anxiety Scale; RSES = Rosenberg Self-Esteem Scale. * *p* < 0.05; ** *p* < 0.01. All analyses were conducted in R (Version 4.5.0).

**Table 5:** Short-term between-group change score comparisons: Δ = T1 − T0.

Measure	Experimental Δ M (SD)	Control Δ M (SD)	U	Z	*p*	r	95% CI (Hodges–Lehmann)
SDS	−7.62 (3.93)	−0.12 (1.13)	0.50	3.31	<0.001 ***	0.83	[−12.00, −4.00]
SAS	−3.50 (2.45)	0.12 (1.13)	5.00	2.86	0.004 **	0.72	[−6.00, −1.00]
RSES	2.12 (0.35)	0.25 (0.71)	60.50	3.30	0.001 **	0.83	[2.00, 2.00] †

Note. Change scores (Δ = post-test minus pre-test) were calculated for each participant. Between-group differences were assessed using the Mann–Whitney U test (N = 16). Negative Δ for SDS and SAS reflects symptom reduction (improvement); positive Δ for RSES reflects self-esteem increase. Effect size r = |Z|/√N (N = 16). † The narrow confidence interval for RSES reflects highly consistent self-esteem gains across participants in the experimental group. Δ values represent the mean of individual within-subject change scores (T1 minus T0) computed separately for each participant prior to averaging, and may therefore differ by 0.01 from the difference between the rounded group means reported in Table 4 due to rounding propagation. ** *p* < 0.01; *** *p* < 0.001. All analyses were conducted in R (Version 4.5.0) applying normal approximation with continuity correction.

**Table 6:** Six-month between-group change score comparisons: Δ = T2 − T0.

Measure	Experimental Δ M (SD)	Control Δ M (SD)	U	Z	*p*	r	95% CI (Hodges–Lehmann)
SDS	−8.50 (4.63)	−0.88 (1.89)	2.00	3.11	0.002 **	0.78	[−13.00, −4.00]
SAS	−5.00 (3.25)	0.50 (2.93)	6.00	2.69	0.007 **	0.67	[−9.00, −2.00]
RSES	2.00 (1.60)	0.00 (1.31)	53.00	2.19	0.029 *	0.55	[0.00, 4.00] ‡

Note. Six-month change scores (Δ = follow-up minus pre-test) were calculated for each participant. Between-group differences were assessed using the Mann–Whitney U test (N = 16). Effect size r = |Z|/√N (N = 16). ‡ The CI [0.00, 4.00] for RSES indicates that the lower bound touches zero, reflecting the modest but consistent six-month self-esteem advantage in the experimental group. * *p* < 0.05; ** *p* < 0.01. All analyses were conducted in R (Version 4.5.0).

**Table 7:** Between-group comparisons at six-month follow-up: absolute scores at T2.

Measure	Experimental T2 M (SD)	Control T2 M (SD)	U	Z	*p*	r
SDS	51.00 (3.12)	55.00 (5.13)	15.00	1.76	0.079 †	0.44
SAS	48.38 (4.87)	52.88 (1.73)	9.50	2.32	0.020 *	0.58
RSES	27.62 (1.77)	24.75 (2.92)	53.00	2.18	0.030 *	0.54

Note. Between-group comparisons at T2 were assessed using the Mann–Whitney U test (N = 16). Effect size r = |Z|/√N (N = 16). Clinical cut-off scores: SDS ≥ 53 (Wang & Wang, 1999); SAS ≥ 50 (Y. Liang et al., 2020). At T2, the experimental group mean for SDS (51.00) and SAS (48.38) fell below the respective clinical thresholds, whereas the control group remained above both thresholds. † *p* < 0.10 (trend-level); * *p* < 0.05. All analyses were conducted in R (Version 4.5.0).

**Table 8:** Sociodemographic and clinical characteristics of all participants (N = 16).

Characteristic	Category	n	%
Age (years)	18–39	3	18.8
	40–59	7	43.8
	≥60	6	37.5
Education	Primary school or below	6	37.5
	Junior secondary	6	37.5
	Senior secondary or above	4	25.0
Marriage duration	<10 years	2	12.5
	10–29 years	5	31.3
	≥30 years	9	56.3
Socioeconomic status	Low	10	62.5
	Moderate	6	37.5
Violence exposure	Psychological violence	16	100.0
	Physical violence	14	87.5

Note. Analytical sample: experimental group (n = 8) and control group (n = 8). A reserve group (n = 11) served as an attrition buffer; no attrition occurred, and reserve group data were excluded from all analyses. Violence exposure categories are not mutually exclusive.

**Table 9:** Overview of qualitative themes identified at six-month follow-up.

Theme	Sub-Theme	Description
Affective and Emotional Change	Relief of depressive symptoms	Participants described improved mood and increased daily engagement.
	Anxiety regulation	Participants reported using sensory memories from the intervention to manage anxiety.
Cognitive and Self-Perception Reconstruction	Identity reconstruction	Participants described a shift from identifying themselves as victims toward survivors.
	Agency and self-efficacy	Participants reported greater confidence in expressing themselves and making decisions.
Behavioral and Social Manifestations	Transfer of coping and creative practices	Participants described continuing to use creative activities and coping strategies in everyday life.
	Interpersonal and social participation	Participants described improved interpersonal interactions and greater community engagement.
Reflections on the Intervention	Meaningful intervention experiences	Participants identified specific activities that continued to support coping and recovery after the intervention.
Barriers and Challenges	Initial resistance and ongoing needs	Participants described early discomfort and expressed a need for continued support.

Note. Themes were generated through inductive thematic analysis (Braun & Clarke, 2006, 2019) of semi-structured follow-up interviews with experimental group participants (n = 8). Coding frequencies are not reported, consistent with recommendations against equating prevalence with thematic importance in qualitative analysis (Braun & Clarke, 2006, 2019).

## Data Availability

The data presented in this study are not publicly available due to ethical and privacy restrictions protecting the identity and safety of women survivors of DV who participated in this study. Restricted access to anonymized data may be granted by the corresponding author upon reasonable request and subject to approval by the Human Research Ethics Committee of Universiti Sains Malaysia (JEPeM-USM).

## Supplementary Materials

The following supporting information can be downloaded at https://www.mdpi.com/article/10.3390/bs16091563/s1, S1: follow-up interview guide; S2: structured observation protocol.

## Abbreviations

The following abbreviations are used in this manuscript:

CI	Confidence Interval
DSM-5	Diagnostic and Statistical Manual of Mental Disorders, Fifth Edition
DV	Domestic Violence
IPV	Intimate Partner Violence
RSES	Rosenberg Self-Esteem Scale
SAS	Zung Self-Rating Anxiety Scale
SD	Standard Deviation
SDS	Zung Self-Rating Depression Scale
T0/T1/T2	Baseline/Post-Intervention/Six-Month Follow-Up Assessment
WHO	World Health Organization
WMA	World Medical Association
JEPeM-USM	Human Research Ethics Committee, Universiti Sains Malaysia
