# Supplementary material for: Canvas of Strength: A Mixed-Methods Pilot Study of a Community-Based Arts Intervention for Women Survivors of Domestic Violence in China
Source: Behav Sci (Basel). 2026 Sep 3;16(9):1563. doi: 10.3390/bs16091563 (PMC13603211; doi:10.3390/bs16091563)
Supplement: Supplementary file 1 [file behavsci-16-01563-s001.zip › behavsci-4440644-supplementary.pdf]

Supplementary Materials

S1:

Survivor Follow-up Interview Guide

Participants: Intervention Group Members (P1–P8)

Timepoint: Six months post-intervention (T2)

Format: Semi-structured Individual Interview

Overall Impressions and Activity Experience

1. Looking back on the group activities, what stands out most strongly in your memory now?

Probes:

- Is this impression mainly related to the activity content, your emotional experience at the time, or interactions with others?
- Has this impression changed or become clearer over time since the activities ended?
- Does this impression relate to a sense of safety, release, or being accepted?

2. Which specific activity segments left the deepest impression on you, and why?

Probes:

- What happened during this activity that made it different from the others?
- Did you notice any bodily sensations (e.g., calmness, tension, relaxation) during or after this segment?
- How long did the feeling or effect last?

3. Were there any particular moments or exercises that contributed most to your personal change? Why were they meaningful to you?

Probes:

- Did this moment lead to a new realization about yourself?
- Did it connect to past experiences or long-standing emotions?
- How did you feel immediately afterward?

4. What aspects of the group environment helped you feel safe and confident enough to participate or express yourself?

Probes:

- Did the facilitator's attitude or way of guiding the activities play a role?
- Did group rules, seating arrangements, or peer responses affect your sense of safety?
- Did your sense of safety increase over time?

5. Were there any activities or exercises that you found unhelpful, uncomfortable, or whose effects felt short-lived? What do you think contributed to this?

Probes:

- Was the difficulty emotional, physical, or related to understanding the task?
- Did your feelings about this activity change later?
- Do you think the discomfort was still meaningful in some way?

Personal Change and Self-Perception

6. What changes, if any, have these activities brought to you? Please describe both positive effects and any difficulties or challenges.

Probes:

- Were these changes immediate or gradual?
- Did any change feel unfamiliar or difficult to adapt to?
- Which change feels most important to you now?

7. Compared with before the activities, has your perception of yourself changed (e.g., confidence, sense of personal value, outlook on the future)?

Probes:

- Do you think differently about your abilities or worth now?
- Are there situations where this change becomes especially noticeable?
- Has the way you speak to yourself internally changed?

8. From the end of the activities until now, what specific changes have you noticed in your daily life or emotional state? Could you give a concrete example?

Probes:

- Are these changes stable or do they fluctuate?
- Can you recall a recent situation where you reacted differently than before?
- Did you intentionally do something differently, or did it happen naturally?

Social Relationships and Coping

9. Since completing the activities, have there been changes in your relationships with family members or friends? Please provide specific examples if possible.

Probes:

- Have there been changes in communication, emotional closeness, or conflict?
- Do you express your needs or feelings more openly now?
- How do others respond to these changes?

10. When you encounter difficulties or stress in daily life, do you recall experiences from the group activities? How do these experiences help you cope?

Probes:

- Are there specific images, exercises, sounds, or feelings that come to mind?
- Do these memories help you calm down, think differently, or take action?
- Can you describe a recent situation where this happened?

Sustainability and Future Suggestions

11. After the activities ended, have you continued to use any arts-based methods (e.g., drawing, journaling) for self-expression? If so, how often? If not, what are the main reasons?

Probes:

- Do you use these methods intentionally or only when emotions arise?
- Have these practices become part of your daily routine?
- What makes it easier or harder to continue them?

12. For future programs of this kind, which activity components do you believe are most important to retain, and which could be improved?

Probes:

- Are there activities you would like to experience more frequently?
- Would changes in duration, frequency, or group size be helpful?
- Are there any skills or techniques you wish had been taught in more detail?

13. Is there any other important experience or feeling related to the group activities

that we have not discussed but that you feel should be included?

S2:
STRUCTURED OBSERVATION PROTOCOL (ENGLISH VERSION)
Observation Rating Manual

The following rating manual was used to structure and quantify observational notes collected during arts-based group sessions. Ratings were assigned retrospectively based on documented behaviours.

Domain 1: Engagement

Score	Behavioural Anchor
0	Mechanical participation; minimal movement; no responsiveness to instructions.
1	Occasional minimal actions or eye contact; largely disengaged.
2	Completes basic requirements; attention brief and easily distracted.
3	Functional participation; follows group pace; occasional distraction.
4	Active participation; initiates ≥2 responses, questions, or feedback.
5	Highly engaged throughout; initiates ≥3 actions and facilitates others.

Domain 2: Interaction

Score	Behavioural Anchor
0	No observable interaction; withdrawn or non-responsive.
1	Rare responses (e.g., nodding, brief words); no initiation.
2	Brief responses when prompted; minimal interaction.
3	Functional interaction (e.g., sharing materials, brief exchanges).
4	Active interaction; initiates conversation or feedback.
5	Supportive interaction; encourages or assists others repeatedly.

Domain 3: Emotional Expression

Score	Behavioural Anchor
0	Flat affect; no discernible emotional expression.
1	Occasional micro-expressions; very brief.
2	Mild emotional response when prompted.
3	Context-appropriate emotional expression.
4	Spontaneous positive emotional expression.
5	Deep emotional resonance; strong affective engagement.

Domain 4: Focus

Score	Behavioural Anchor
0	Extremely distracted; delayed or absent responses.
1	Brief focus; frequent distraction.

2	Intermittent focus; easily disrupted.
3	Mostly focused with occasional distraction.
4	Sustained focus throughout most of the session.
5	Fully absorbed; rapid and consistent responses.

Domain 5: Creativity

**Score**	**Behavioural Anchor**
0	Mechanical task completion; no personal input.
1	Minimal experimentation; imitation-based.
2	Limited personal attempts; low originality.
3	Functional creativity within task framework.
4	Clear personal style; exploration of materials or ideas.
5	Highly original expression; inspires or influences others.

Domain 6: Stress

**Score**	**Behavioural Anchor**
0	Emotionally flat; low arousal, low engagement.
1	Mild tension (minor fidgeting); task unaffected.
2	Recurrent mild anxiety or discomfort.
3	Observable anxiety (e.g., rigid posture, restricted speech).
4	High stress; withdrawal, avoidance, or somatic signs.
5	Extreme stress response; crying, trembling, or need for support.

Structured Observation Rating Sheet (Per Session)

**Session ( )**	**Participant ID**	**Engagement**	**Interaction**	**Emotion**	**Focus**	**Creativity**	**Stress**	**Total Score**	**Observer Notes**
	**1**								
	**2**								
	**3**								
	**4**								
	**5**								
	**6**								
	**7**								
	**8**								

Note.Total Score may be calculated for descriptive comparison across sessions; no clinical cutoff was applied. One rating sheet was completed for each session based on extracted observational notes.
